# Approaches to engineer stability of beetle luciferases

**DOI:** 10.5936/csbj.201209004

**Published:** 2012-10-09

**Authors:** Mikhail I. Koksharov, Natalia N. Ugarova

**Affiliations:** a Department of Chemical Enzymology, Faculty of Chemistry, Lomonosov Moscow State University, Moscow, 119991, Russia

**Keywords:** Firefly luciferase, Bioluminescence, protein engineering, directed evolution, thermal stability

## Abstract

Luciferase enzymes from fireflies and other beetles have many important applications in molecular biology, biotechnology, analytical chemistry and several other areas. Many novel beetle luciferases with promising properties have been reported in the recent years. However, actual and potential applications of wild-type beetle luciferases are often limited by insufficient stability or decrease in activity of the enzyme at the conditions of a particular assay. Various examples of genetic engineering of the enhanced beetle luciferases have been reported that successfully solve or alleviate many of these limitations. This mini-review summarizes the recent advances in development of mutant luciferases with improved stability and activity characteristics. It discusses the common limitations of wild-type luciferases in different applications and presents the efficient approaches that can be used to address these problems.

## Introduction

Firefly luciferase catalyzes the two-step oxidation of firefly luciferin in the presence of ATP, Mg^2 +^, and molecular oxygen which is accompanied by the emission of visible light [1, 2]. This reaction is the same for all bioluminescent beetles but historically the enzyme from *Photinus pyralis* fireflies was the first to be extensively studied, so all representatives of this enzyme family are often called “firefly luciferases”. The peak of the light emission varies from 538 to 623 nm for the enzymes from different species or for the mutant luciferases but the yellow-green bioluminescence is the most common [3]. Beetle luciferases demonstrate a notable quantum yield (45-60%), which is the highest among bioluminescent systems [6]. Firefly luciferases show bright bioluminescence, low background signal, high catalytic efficiency, substrate specificity and high sensitivity to ATP. This makes them a widely used tool in a variety of *in vitro* and *in vivo* applications: in ATP-related assays from direct ATP measurements to estimation of bacterial contamination and pyrosequencing [4, 5], in *in vivo* molecular imaging and as a genetic reporter in molecular biology [6–8]. This enzyme was also shown to be a promising tool for molecular sensing of protein-protein interactions and different analytes [9–11], in analytical assays based on real time monitoring of polynucleotide amplification [12] and a label for immunoassays [13].

Many novel beetle luciferases with promising properties have been reported in the recent years [14–16]. Some of them were developed into *in vivo* reporters which are superior to the commonly used *P. pyralis* luciferase (Ppl) [17]. However, the applications of wild-type (WT) beetle luciferases are often limited by insufficient stability of these enzymes at elevated temperatures above 30°C. Therefore, the development of thermostable forms of luciferase is often required [18, 19] and this problem arises for the recently cloned promising enzymes. For example, the most commonly used Ppl looses half of its activity within 15 min at 37°C and some of the newly cloned luciferases inactivate even faster [19]. Thermal stability of luciferases is most crucial for *in vitro* assays: immunoassays and pyrosequencing are usually conducted at 37°C [5] and assays based on polynucleotide amplification require luciferase to be stable at least at 50°C (preferably at temperatures >60°C) [12]. This problem is less pronounced in common *in vivo* applications since the *in vivo* half-life of Ppl is around 3-4 h at 37°C in mammalian cells [20], which is usually sufficient to monitor gene expression and for molecular imaging. However, more stable luciferases significantly improve the *in vivo* bioluminescence signal and provide more sensitive detection [19, 21]. If intracellular processes are needed to be monitored at higher *in vivo* temperatures then the thermostability becomes crucial since Ppl inactivates within 5-20 minutes *in vivo* at 40-45°C in eukaryotic cells [22, 23]. High thermostability of enzyme can also be highly beneficial for evolving other types of stability and new enzyme functionalities [24] such as a recent work on changing luciferase substrate specificity [25] or the popular trend to develop multi-color luciferases [26].

Another problem that often needs to be addressed is denaturation or inhibition of firefly luciferase at conditions of a particular assay. For example, in hygiene monitoring the inhibition from the extractants used for releasing intracellular ATP is a common problem [4]. The activity of luciferase during monitoring of *in vivo* bioluminescence can be affected by various intracellular factors including pH, proteases, pyrophosphate, reactive oxygen species, etc [27–29]. The latter can affect not only the sensitivity of detection but the interpretation of results as well.

A large number of works have been reported that describe the development of mutant luciferases with enhanced properties that showed improved stability towards the action of temperature and other factors. Like with the general field of protein engineering these works followed structure-based rational design approach [30] or random mutagenesis / selective screening approach [31]. Both strategies gave many successful examples of luciferase stabilization. However, the random mutagenesis approach can be very efficient in case of luciferase because colony libraries of mutant luciferases can be rather easily screened for activity (emitted light) in the presence of different factors which is often quite cumbersome for many other enzymes [31, 32].

This mini-review discusses the recent results in engineering stable and active beetle luciferases, describes the types of stability required in different applications and compares the strategies that can be efficiently used to achieve a desirable level of luciferase stability. The major enhanced variants of beetle luciferases discussed here are summarized in the Supplementary Table 1.

## Thermal stability of wild-type beetle luciferases

**Figure 1:**
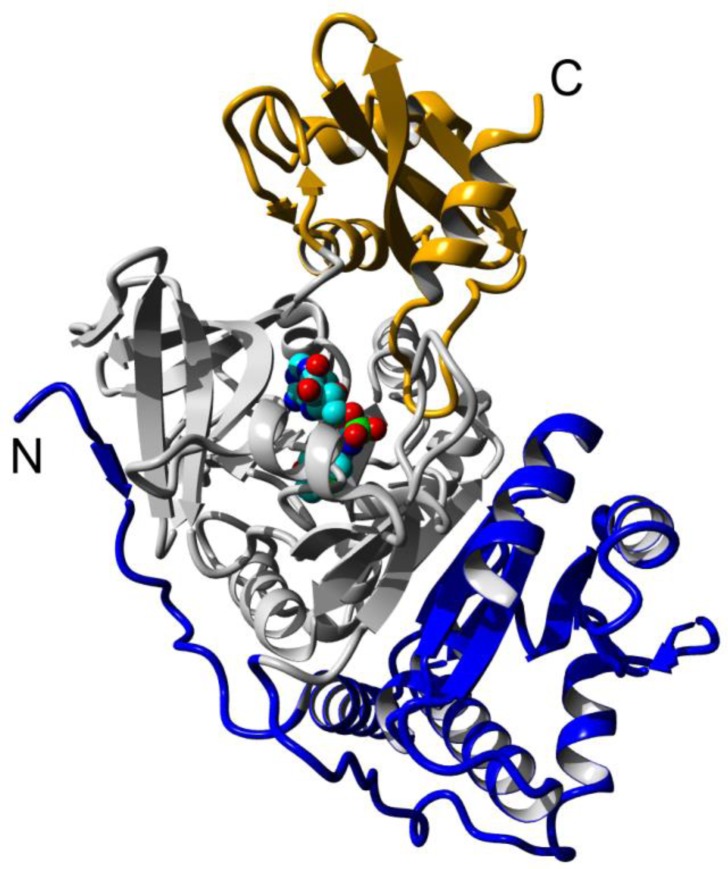
**Structure of beetle luciferases (*L. cruciata* firefly luciferase in complex with DLSA [42])**. Subdomains A, B and C are depicted in blue, grey and orange, respectively

Firefly luciferases can be relatively stable *in vitro* in solution at low temperature in the presence of stabilizing compounds, though at low concentration without protective additives up to 99% of the enzyme can be lost due to the protein adsorption on the container surface [33]. However, even in the presence of stabilizing compounds Ppl luciferase inactivates within 6-20 minutes at 37-42°C [18, 34, 35]. Similar stabilities were reported for most other beetle luciferases [26, 36]. The inactivated luciferase is almost unable to restore activity after cooling and usually aggregates [22]. It can be effectively reactivated only in the presence of different chaperone systems [37]. The detailed mechanism of luciferase inactivation in solution is still unknown and may vary for enzymes from different species. The knowledge of the inactivation and unfolding mechanism is necessary for the definite prediction of mutations that would increase thermostability; otherwise, the particular stabilization approach may be found not efficient because of the different factors defining the thermostability [30]. In several works different unfolding intermediates of Ppl were analyzed [38, 39]. It was shown that *Luciola mingrelica* luciferase undergoes two-step inactivation with a homodimer dissociation step [40] unlike the Ppl enzyme. The crystal structures of luciferase [41, 42] show that this enzyme consists of a big N-domain (1-436 aa) and a small C-domain (∼443-544 aa) which are connected by a flexible loop. The N-domain is further composed of two distinct subdomains: A (1-190) and B (191-436) stacked together via a strong hydrophobic interface (Fig. 1).

Regarding this structure, the most interesting were the results of Frydman *et al* [38] who had investigated the unfolding of Ppl by chemical denaturation with subsequent protease treatment. They have shown that the middle subdomain “B” (192-435 aa) is significantly less stable that the other two and that it is the first to unfold under denaturing conditions. It may be assumed that the intrinsically low stability of the second subdomain is the “bottleneck” that determines the stability of the whole protein. Therefore, it is not surprising that almost all stabilizing mutations reported in the literature are located in this subdomain or on the interface between the middle subdomain with the first and third subdomains. It is further confirmed by our recent finding [43] that the structurally destabilizing mutation E457K in C-domain doesn't affect the thermostability of the WT luciferase but causes the 3-fold decrease in stability of the highly thermostable mutant [44] stabilized by four mutations in the middle subdomain. Thus, the effect of the deleterious mutation E457K in the third subdomain is only noticeable when the second subdomain is sufficiently stabilized. The similar picture was observed for thermolysin-like protease whose inactivation is governed by the unfolding of the N-terminal domain [30].

## Rational design of thermostable luciferases

Relative improvements in stability at 37°C can be achieved by the addition of stabilizing compounds [5, 45, 46] but the effect is limited and the resultant solution may be incompatible with the particular application. The mutagenesis approach which increases the intrinsic stability allows to achieve much higher stabilization without changing the assay conditions. Before the 3D-structure of luciferase was obtained the only viable strategy to increase the thermostability was random mutagenesis. Several stabilizing mutations were identified by this approach in the early 1990s: the substitution of A217L in *Luciola cruciata* and *Luciola lateralis* luciferases [47, 48] and the substitutions T214A, I232A, F295L, E354K in Ppl [34]. The identified positions were further extensively analyzed by site-directed mutagenesis to identify the most efficient substitutions. The major part of the following work was focused on developing thermostable multi-point mutants that would include these and other previously identified positions. Branchini *et al* have constructed a 5-point mutant of Ppl (T214A/A215L/ I232A/F295L/E354K) which showed a 44-fold improvement of half-life from 15 min to 11.5 h at 37°C. These mutations were further combined with the green and red emitting mutants to give a thermostable mutant pair for the dual-color imaging [18, 26]. Even more striking example was reported by Murray *et al* [49] who have combined almost all previously known single thermostabilizing mutations in the highly stable 12-point mutant of Ppl. This mutant had a half-life of 15 min at 55°C whereas WT luciferase inactivates within seconds at these conditions.

However, the mutant possessed only 15% of the original activity which shows one of the downsides of this approach: in case of combining many individual mutations it may require additional extensive and laborious analysis by site-directed mutagenesis to identify the mutations which will retain the high activity in addition to high stability. Another limitation of this approach is that the mutations obtained for one particular enzyme often can not be directly applied to another homologous enzyme. For example, the mutation A217L was discovered in *L. cruciata* luciferase and was successfully applied to *L. lateralis* and *P. pyralys* luciferases to give highly active and stable mutants. However, the same mutation caused the loss of activity in *Luciola parvula* luciferase [35]. Likewise, the mutation E354R increased thermostability of Ppl but the corresponding mutation E356R did not affect the stability of *L. parvula* luciferase [35].

In that case a comparative analysis of the selected residue microenvironment may be used to efficiently implement such problematic mutations. In our laboratory we have compared the microenvironment of A217 in *L. mingrelica* luciferase with that of *L. cruciata* and *P. pyralis* luciferases and identified 2 additional mutations (G216N, S398M) that should be introduced along with A217L to obtain a thermostable triple mutant without significant decrease in catalytic activity [50]. The double mutant G216N/A217L demonstrated 18-fold increase in thermostability but the activity was only 10% of WT, and the third mutation S398M was necessary to restore the catalytic properties.

After the structure of firefly luciferase became known, several classic structure-based rational protein design approaches [30] were applied to firefly luciferase to increase its thermostability. For example, hydrophilization of the protein surface was successfully used in case of Ppl [51]. In this work the authors have chosen five bulky solvent-exposed hydrophobic residues that are not conservative and do not form any secondary interactions. These residues were mutated into different hydrophilic residues and screened for the thermostability. The best substitutions were combined into the 5-point mutant (F14R/L35Q/V182K/I232K/F465R) which showed greatly improved pH-tolerance and stability up to 45°C without any decrease in activity or catalytic efficiency. Recently, the similar approach was successfully used for *Lampyris turkestanicus* luciferase where mainly the same surface residues were mutated to arginine [52].

The opposite approach is the hydrophobization of the protein globule can also be a quite efficient strategy since hydrophobic protein core is the major determinant of the protein stability [53]. The thermostabilizing mutations identified to this date confirm that this method can be used for beetle luciferases with a good success rate. In this approach buried non-conservative polar residues are mutated to hydrophobic ones and small internal hydrophobic residues can be mutated to larger ones if the latter would fill in an internal cavity. The results of site-directed mutagenesis [48] and the 3D-structures of luciferase show that the substitutions of the previously mentioned residue A217 by valine, leucine and isoleucine are the most efficient because they fill in the internal cavity thus improving the hydrophobic packing of the protein globule. Analysis of the structure of *L. mingrelica* luciferase shows that there are only four buried polar residues that are non-conservative in luciferases from fireflies and are often substituted to hydrophobic groups: R211, S364, S398 and S404. The mutations for the two of them to hydrophobic residues (R211L, S364A, S364C) were shown to increase thermostability [44], while the mutation S398M did not affect the overall thermostability but increased the local conformational stability [50]. The buried polar residue S118 is conservative in most firefly luciferases but changes to valine in click-beetle luciferases. The mutation S118C were shown to increase thermostability 1.5-fold at 42°C [44].

Among the surface residues, cysteines can have a detrimental effect on enzyme storage stability leading to oxidative cross-linking and aggregation. *L. mingrelica* luciferase contains eight cysteine residues that don't form any disulfide bonds and three of them are conservative. This enzyme requires the presence of a reducing agent such as dithiothreitol in the storage buffer; otherwise, it gradually inactivates by more that half within several days at 0-4°C. It was shown that the mutation of the non-conservative C146 to serine increases the thermostability 1.3-fold at 42°C [54] and eliminates the need for the dithiothreitol in the storage buffer [44].

One of the most efficient approaches to stabilize protein is a covalent binding of two parts of its structure by disulfide bond [30]. Hosseinkhani *et al* have applied this strategy to Ppl [55, 56] by introducing 5 different disulfide bonds. The degree of stabilization varied from mild to several-fold increase of thermostability which is within the range of some single mutations like A217L [47, 48] or E354K [57]. The disulfide bonds A103C-S121C and L306C-L309C conferred the highest stability but caused the 20% or 95% decrease in activity, respectively. On the other hand, the introduction of the disulfide bonds C81-A105C and A296C/A326C improved the activity 2-fold and 7-fold, respectively.

One of the factors that reduce *in vivo* half-life of luciferase is its sensitivity to proteases. The folded enzyme is relatively resistant to proteolysis but elevated temperatures result in partial enzyme unfolding which leads to higher accessibility of proteolytic sites [38]. Therefore, the proteolytic resistance is usually increased along with the overall or local conformational stabilization [38]. Another approach is the elimination of protease recognition sites; though, in case of luciferase some of them are located in the active site [58]. Riahi-Madvar and Hosseinkhani have employed this strategy [59] and achieved up to 5-fold increase in half-life for the mutants R213M and R337Q under trypsin digestion conditions. Such mutant luciferases may be beneficial as *in vivo* gene reporters.

## Directed evolution of thermostable luciferases

As it was mentioned above, the use of site-directed mutagenesis may require an extensive and laborious analysis of the proposed positions and the results obtained for one luciferase are not always transferable to another. On the other hand, the beetle luciferases have a distinct advantage that they can be easily screened for *in vivo* bioluminescence activity on the level of *E. coli* colonies [60]. This fact makes directed evolution approach the most promising of the evolving various properties of luciferase. In this strategy multiple consecutive cycles of random mutagenesis and screening are used for an incremental increase of the required property of an enzyme. The approach of directed evolution is especially efficient if the simple screening strategy is available like in the case of firefly luciferase. In such case it can be superior to rational protein design; otherwise, the screening procedure can be very costly and require extensive labor [31, 32]. However, there is only one example when this approach was used to increase the thermostability of firefly luciferase. The most stable firefly luciferase to date is a mutant of *Photuris pennsylvanica* luciferase obtained by directed evolution (“Ultraglow luciferase”), which contains 28 substitutions and shows a half-life of 27 h at 65°C [61, 62]. In this case a sophisticated automatic robotic system was used for the screening procedure allowing to simultaneously monitor several kinetic characteristics. Possibly, the complexity and cost of this technique limited its wide application. The other disadvantage that was reported for this highly stable mutant is low activity which is only 4% compared with WT Ppl [49].

**Figure 2:**
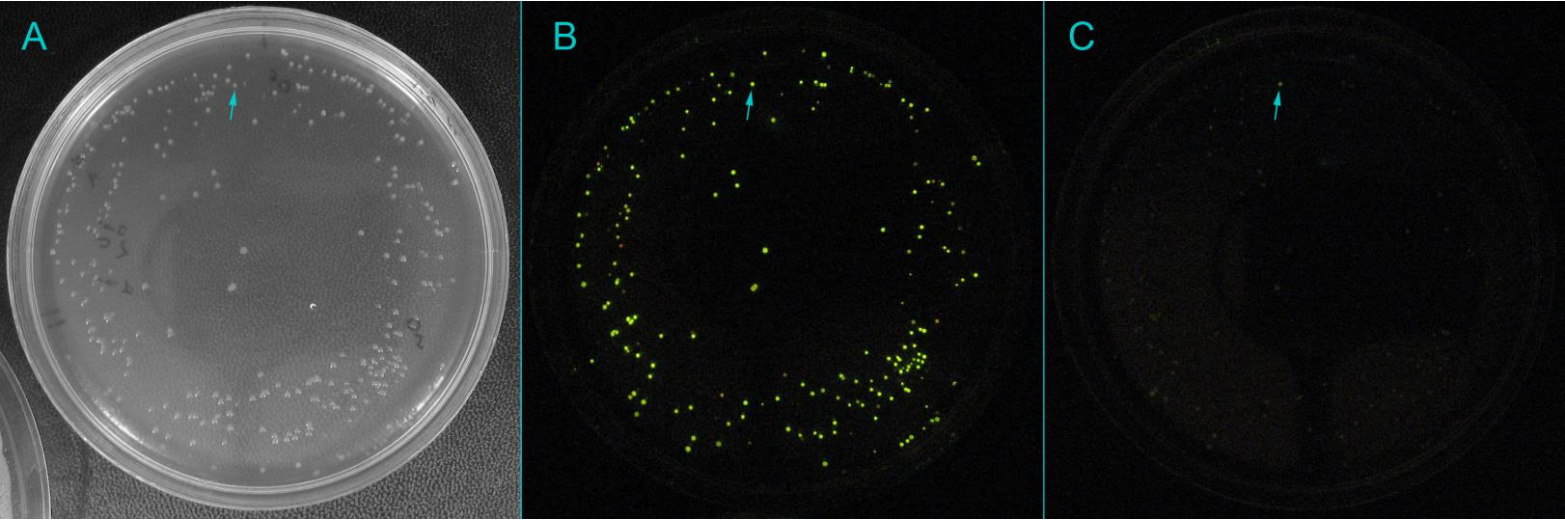
**Typical non-lethal *in vivo* screening of the 90 mm plate (A) with mutant *E. coli* colonies for thermostability. *In vivo* bioluminescence before (B) and after (C) incubation of the plate at 50°C [44]**. The thermostable mutant is marked by the arrow.

Recently, we have employed a much more simple but efficient *in vivo* screening strategy to evolve a thermostable form of *L. mingrelica* luciferase without compromising its activity. The *in vivo* bioluminescence of large and dense libraries of *E. coli* colonies can be easily detected photographically without killing the cells. On the other hand, *E. coli* cells survive heating up to 55°C. This allowed us to identify thermostable mutants by simple non-lethal *in vivo* screening of *E. coli* colonies that produce mutant luciferases (Fig. 2). *E. coli* cells remain viable after the screening and can be picked directly from the same plate which eliminates the need in using replica plates. Thus, with this screening strategy each round of screening could be carried out in a simple and rapid manner. Four consecutive cycles of directed evolution resulted in the mutant 4TS, which showed 66-fold improvement of stability at 42°C from 9 min to about 10 h. It also demonstrated 1.9-fold increase in activity, 6.7-fold improvement of *K*_*m*_ for ATP and increased activity at high temperatures [44]. It retains 70% of activity *in vitro* after two days of incubation at 37°C, which is sufficient for most common applications. This mutant is one of the most stable mutant luciferases and only surpassed by the thermostable mutant of *L. lateralis* [47], 12-point mutant of Ppl [49] and 28-point mutant of *P. pennsylvanica* [62] luciferases, though the first mutant shows mild [63] and the latter two show substantial decrease in activity [49]. This screening strategy is the simplest among reported in the literature and can potentially be used to efficiently increase thermostability of other beetle or non-beetle luciferases. Since the bioluminescence is detected before and after the heating step it makes a decrease in activity unlikely for the selected mutants.

## Engineering destabilized luciferases

High stability of luciferase is usually beneficial in *in vitro* assays as well as in *in vivo* reporter applications. However, in some cases it is desirable to have either a luciferase reporter with short *in vivo* half-life or intrinsically unstable luciferase. The *in vivo* half-life of the WT Ppl is 3-4 h in mammalian cells which makes it difficult to detect short-term changes in gene expression, especially the decreases owing to the accumulation of residual luciferase [20]. The addition of the proteolytic “PEST” sequence from mouse ornithine decarboxylase decreased the functional half-life of luciferase to 0.84 h compared with 3.68 h for the WT enzyme [20]. However, even with the use of such destabilizing sequence highly thermostable luciferases can pose a problem. For example, a more thermostable beetle luciferase showed a small but noticeable phase shift compared with Ppl when monitoring circadian oscillations of gene expression [64], though both proteins were fused to the PEST signal. Recently, a system to monitor a particularly short expression processes (rapid bursts in mammalian gene transcription) was reported which uses a short-lived messenger RNA coding a short-lived PEST-fused firefly luciferase [65].

In contrast to the use of a proteolytic signal which does not affect the internal stability of luciferase, a set of structurally destabilized mutants of firefly luciferase was recently developed [66]. The destabilization was achieved through the mutations R188Q and R261Q outside of the substrate-binding pocket which disrupt two conservative hydrogen bonds that contribute to the connection between the second and the first subdomains of luciferase (Fig. 1). These destabilized mutants require the presence of chaperones for the efficient folding and maintaining of the active state and can serve as reporters of cellular proteostasis capacity. They were successfully used as sensors of intracellular proteomic stress at temperatures 20-37°C, particularly in *Caenorhabditis elegans* which grows at 20°C [66].

## Engineering resistance towards other denaturing and inhibiting factors

The structural basis for stability to factors other than temperature is much less clear than thermostability, so random mutagenesis approach is usually the most efficient in evolving this type of resistance [31, 32]. Similar to thermostability, the ease of photographic detection of bioluminescence activity makes colony-based screening of mutant colony libraries a very promising approach. The most straightforward scheme [67–69] includes lysis of colonies on a filter membrane, subsequent treating the lysed colonies with a buffer containing the denaturing or inhibiting factor for the required time followed by the photographic detection of bioluminescence.

The inactivation of luciferase by denaturing or inhibiting factors and compounds often becomes a limiting problem, especially for *in vitro* ATP-related assays. However, the type of required stability is usually specific to a particular assay, so such mutants have a more narrow application than thermostable luciferases. For example, intracellular ATP levels reflect cell viability and luciferase-based ATP-assays can be used to assess cytotoxicity of industrial chemicals [4]. However, these chemicals themselves usually inhibit the reaction affecting the assay sensitivity. Kim-Choa *et al* have used random mutagenesis to identify mutants of Ppl resistant to low concentrations of chloroform [70]. The screening scheme included the primary step of *in vivo* selection of mutant colonies on nitrocellulose membranes followed by secondary *in vitro* screening. After two rounds of mutagenesis the mutant S239T/D357Y/A532T was obtained which showed 3-fold higher activity (90%) in the presence of 0.5% chloroform compared with the WT luciferase. The mutant also showed increased stability in the presence of other organic compounds such as ethanol, hexane, toluene, etc [70]. The mutant was also more active in the presence of detergents such as Triton X-100 and SDS [71].

Hygiene monitoring and bacterial biomass estimation assays require the extraction and precise measurement of the intracellular ATP. The crucial step is quick ATP extraction that should preserve the native ATP concentration [4]. The different organic compounds such as trichloric acid, benzalkonium chloride (BAC), dimethyl sulfoxide are the most efficient but again strongly decrease the luciferase activity. Hattori *et al* have used random mutagenesis followed by *in vitro* screening of ∼1000 active mutants for their resistance to 0.1% BAC [63]. The mutant E490K was identified which was resistant to 8-14% higher concentration of BAC compared with the WT enzyme. Therefore, the development of luciferase mutants resistant to different extractants and organic inhibitors is a promising task that can significantly improve ATP-related luciferase assays.

Another promising direction of research that was not yet addressed in the literature is the development of luciferase resistant to intracellular inactivating factors. For example, variations in the intracellular pH and other factors can dramatically affect *in vivo* luciferase activity and interpretation of the data [27, 28]. Recently, a Ppl-based sensor was developed for monitoring intracellular H_2_O_2_ [72]. However, another report have shown that Ppl is sensitive to reactive oxygen species (ROS) (including H_2_O_2_) and that its *in vivo* activity can be substantially altered in studies where ROS levels become elevated which can potentially lead to ambiguous or misleading findings [29].

## Conclusions

The analysis of literature shows considerable achievements in engineering stability of beetle luciferase. Several highly thermostable mutants are now available that can suit the needs of most bioluminescence assays. Many specific positions have been identified that can be generally used to increase thermostability of new promising luciferases. Hydrophilization of non-conservative hydrophobic surface residues and hydrophobization of non-conservative buried polar residues seem to be an efficient general rational approach to increase the thermostability of luciferases. However, the ease and efficacy of thermostability screening of beetle luciferase make the directed evolution approach the most efficient to rapidly evolve thermostable mutants without decrease in activity. Therefore, if the stability of any wild-type luciferase needs to be increased, the directed evolution approach is the first to try. After the highly thermostabilized mutant is evolved, site-directed mutagenesis can be used to further optimize this enzyme: by finding the most efficient substitutions for the identified positions, by adding other known thermostabilizing mutations and by removing the mutations with undesirable effects on other properties, such as, for example, small color-shifts [26].

The development of luciferases, that are resistant to the action of assay-specific *in vitro* or *in vivo* inactivating compounds and factors, still remains a challenging task and a promising direction of research which can significantly enhance the sensitivity and reliability of many luciferase-based applications.

## Supplementary Material

Approaches to engineer stability of beetle luciferases
