# Supplementary material for: Approaches to engineer stability of beetle luciferases
Source: Comput Struct Biotechnol J. 2012 Oct 9;2:e201209004. doi: 10.5936/csbj.201209004 (PMC3962189; doi:10.5936/csbj.201209004)
Supplement: Approaches to engineer stability of beetle luciferases [file CSBJ-2-e201209004_SM0001.pdf]

SUPPLEMENTARY MATERIAL

Approaches to engineer stability of beetle luciferases

Mikhail I. Koksharov*, Natalia N. Ugarova

Department of Chemical Enzymology, Faculty of Chemistry, Lomonosov Moscow State University, Moscow, 119991, Russia. E-mail: mkoksharov@gmail.com

Table S1. Key enhanced mutant variants of beetle luciferases described in this mini-review

Luciferase	Properties of the mutant	Mutagenesis approach[1]	Ref.
Photuris pennsylvanica	28-point thermostable mutant: half-life of 27 h at 65°C;<br>4% activity compared with WT *P. pyralis* luciferase [49]	Directed evolution	[61,62]
Photinus pyralis	The set of single mutants with increased thermostability: A215L, T214A, I232A, F295L, E354K	Random	[34, 57]
	4-point thermostable mutant T214A/I232A/F295L/E354K:<br>1.9-5.6-fold increase in stability at 35-40°C;<br>56% activity compared with WT	Site-directed (SD)	[34]
	5-point thermostable mutant T214A/A215L/ I232A/F295L/E354K:<br>44-fold increase in half-life at 37°C: from 15 min to 11.5 h;<br>90% activity compared with WT		[18,26]
	12-point thermostable mutant: half-life of 15 min at 37°C;<br>15% activity compared with WT		[49]
	5-point thermostable mutant F14R/L35Q/V182K/I232K/F465R:<br>6-fold increase in half-life at 43°C: from 5 min to 33 min;<br>82% activity compared with WT	Rational design, SD	[51]
	The set of mutants containing engineered disulfide bonds:<br>moderate thermostabilization or significant increase in activity for some of the variants	Rational design	[55,56]
	The mutants with reduced sensitivity to proteases: R213M and R337Q		[59]
	Structurally destabilized mutants for monitoring *in vivo* folding and proteomic stress: R188Q, R261Q and R188Q/R261Q		[66]
	The 3-point mutant S239T/D357Y/A532T with increased resistance to small amounts of chloroform and other organic solvents	Random	[70, 71]
Lampyris turkestanicus	5-point thermostable mutant F14R/L35Q/V182K/I232K/F465R:<br>Q35R/I182R/I232R/E354Q/insertion-R356;<br>7.4-fold increase in half-life at 35°C: from 2.6 min to 19.2 min	Rational design, SD	[52]
Luciola cruciata	Thermostable mutants T217L, T217I, T217V:<br>7.4-fold increase in half-life at 50°C: from 3.8 min to 28 min	Random	[48]
Luciola lateralis	The thermostable mutant A217L:<br>19-fold increase in half-life at 50°C: from 6.5 min to 125 min<br>70% activity compared with WT [63]	SD	[47]
	The mutant E490K with increased resistance to benzalkonium chloride	Random	[63]
Luciola (Hotaria) parvula	A217L: 0.074% of WT activity;<br>E356R: no significant influence on thermostability;<br>E356R/V368A: 12-fold increase in half-life at 45°C: from 18 min to 210 min	SD	[35]
Luciola mingrelica	7-point thermostable mutant 4TS:<br>155-fold increase in half-life at 50°C: from 50 min to 129 h;<br>158-fold increase in half-life at 45°C: from 13 min to 33.4 h;<br>20-fold increase in half-life at 50°C: from 2.2 min to 44 min;<br>190% activity compared with WT	Directed evolution	[44]
	5-point thermostable mutant G216N/A217L/S398M:<br>21-fold increase in half-life at 45°C: from 13 min to 280 min;<br>70% activity compared with WT	Rational design	[50]
	The mutant C146S: 1.3-fold increase in thermostability at 42°C and	Rational	[44,54]

	increased oxidation stability	design	
Phrixothrix hirtus	The mutants I212L/N351K, I212L, I212L/S463R:<br>Increased thermostability in cell extracts and cells at 37°C	SD	[19]

[1]Abbreviations: "random" – random mutagenesis; SD – site-directed mutagenesis (of the previously identified positions in luciferase).
